# Therapeutic management in a pregnant patient with acute ischemic stroke in the revascularization window: A case report

**DOI:** 10.3892/mi.2026.296

**Published:** 2026-01-11

**Authors:** Antonia Ioana Vasile, Amelia Damiana Trifu, Cristina Nica, Simona Trifu

**Affiliations:** 1 Doctoral School, Carol Davila University of Medicine and Pharmacy, Bucharest 050474, Romania; 2 Department of Neurology, University Emergency Hospital of Bucharest, Bucharest 050098, Romania; 3 Medical-Military Institute, Carol Davila University of Medicine and Pharmacy, Bucharest 010919, Romania; 4 Department of Neurosciences, Carol Davila University of Medicine and Pharmacy, Bucharest 050474, Romania

**Keywords:** pregnancy, thrombolysis contraindications, thrombectomy indications, contrast in pregnancy, minor thrombophilia

## Abstract

Pregnancy is considered a relative contraindication for thrombolysis. Moreover, mechanical thrombectomy should be considered for pregnant patients, as it would be indicated in non-pregnant patients. The present study describes the clinical case of a 29-year-old female full-term pregnant patient who presented to the emergency room with a sudden onset symptomatology characterized by pronunciation disorder and motor impairment in the right arm with numbness at the same level, and with a National Institutes of Health Stroke Scale score of 7 points. The patient presented in the revascularization window, and the series of acute medical interventions were as follows: Clinical examination, native head computed tomography (CT) scan, emergency cesarean section, CT angiography of the cerebral arteries, and, eventually, mechanical thrombectomy. A brain MRI revealed a hyperintense lesion in diffusion sequence with low apparent diffusion coefficient correspondence at the frontal level of the left side, affecting the middle and precentral gyrus. A transesophageal ultrasound revealed a small patent foramen ovale with a risk of paradoxical embolism score of 8 points. Usual thrombophilia laboratory test results were negative; however, a homozygous methylenetetrahydrofolate reductase gene mutation and a heterozygous positive plasminogen activator inhibitor-1 gene mutation were detected. On the whole, the present case report emphasizes the importance of evaluating inherited genetic thrombophilia and PFO in young patients suffering a stroke. Moreover, the need for the psychological and psychiatric evaluation for possible reactive depression, anxiety and burnout in young patients suffering a stroke, and particularly in the peripartum period, is highlighted.

## Introduction

During pregnancy and the peripartum period, there are changes in the coagulation system, which may lead to a physiological hypercoagulable state (1). Hemodynamic factors and venous stasis are certain factors that increase the risk of suffering an ischemic stroke in pregnant women (2). These changes predispose the pregnant patient to higher rates of prothrombotic events, such as deep vein thrombosis, superficial vein thrombosis, pulmonary thromboembolism or ischemic stroke (3). Although pregnancy generally increases the risk of developing thrombosis, the highest risk is during peripartum period, which is ≤12 weeks following delivery (4). Statistically, the majority of stroke cases occur following delivery (50%) and in the peripartum period (40%), with the lowest probability (10%) in the antepartum period (5).

Imaging in pregnant patients is a diagnostic dilemma due to radiation exposure (6). Certain factors that influence the potentially harmful effects of radiation from CT scans for pregnant patients are as follows: Gestational age, the total dose of radiation that the patient is exposed to, fetal exposure to radiation, and the need for the administration of contrast agents. Whilst fetal exposure to radiation from head CT scans is extremely low, the radiation dose is markedly higher during head CT scans with intravenous contrast due to multiple additional scans (7). Therefore, magnetic resonance imaging (MRI) is the recommended imaging method, using non-contrast-enhanced time-of-flight magnetic resonance angiography (6).

Previous studies recommend that thrombolysis should be considered when the expected benefits of treating life-threatening or debilitating stroke outweigh the risk of uterine bleeding (8,9). However, at present, to the best of our knowledge, there are no randomized controlled trials that have evaluated pregnant patients with thromboembolism. A recent review highlighted that the risk associated with thrombolytic agents in pregnant patients appears reasonable when balanced with the risk associated with a disease that places the life of the patient in danger (1). In addition, it has been reported that the complications rate of thrombolysis in pregnant patients is not higher than that of non-pregnant patients (1,3). In a previous review article, of the 30 pregnant patients suffering a thrombolysed stroke included in the review, the main complications described were the following: Major bleeding (n=1), maternal mortality due to arterial dissection (n=1), and spontaneous abortions (n=3) (1). However, pregnancy is still considered a relative contraindication for thrombolysis (1).

Certain case studies have suggested that mechanical thrombectomy can be performed safely during pregnancy, particularly for pregnant patients who present with severe stroke [demonstrated by high National Institutes of Health Stroke Scale (NIHSS) scores] (6,9). The patients in these cases experienced excellent recovery and there was no impact on the infants (6). Moreover, it is widely accepted that mechanical thrombectomy in pregnant patients is technically feasible with acceptable rates of recanalization and maternal outcomes and fetal outcomes (8). Mechanical thrombectomy using the Penumbra system appears to be a safe and effective treatment option for acute ischemic stroke during pregnancy when thrombolysis is unsuitable (10). Therefore, mechanical thrombectomy should be considered for pregnant patients as would be indicated in non-pregnant patients (8).

## Case report

A 29-year-old female patient, who was 38 weeks pregnant, presented at the Emergency Room of University Emergency Hospital of Bucharest, Romania, on a Sunday, in June 19, 2023. This was the patient s first pregnancy, with no previous spontaneous abortions. A cesarean section was scheduled for the following day as the patient had two placentas. The patient was a smoker (10 pack years), with no history of gestational diabetes, preeclampsia, eclampsia or hypertension.

The patient was admitted to the hospital for sudden onset symptomatology characterized by a motor deficit and numbness of the right upper limb associated with pronunciation disorder. The neurological clinical examination upon admission revealed the following: Mild-moderate dysarthria (slurred speech, but understandable); minor paralysis (flat nasolabial fold, smile asymmetry); right brachial mono paresis 3/5 on the Medical Research Council (MRC) scale; mild-moderate sensory loss on the right upper arm; and ataxia in right upper arm. The patient had an a NIHSS score of 7 points, and the score was disabling, in that the patient was a 29-year-old with motor deficits in the dominant hand and who was due to have a newborn the following day. Moreover, the patient presented in the revascularization window at ~30 min from symptomatology onset, as a thrombolysis code.

**Figure 1:**
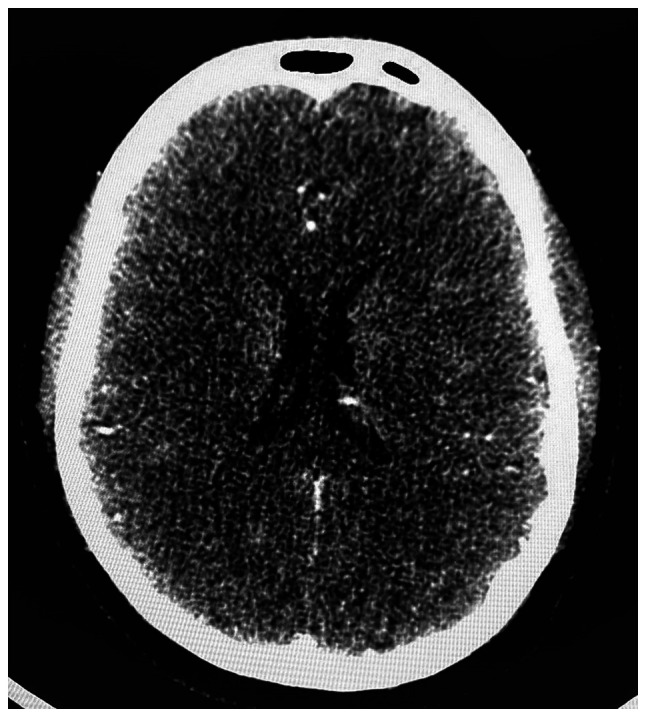
Native head computed tomography scan.

**Figure 2:**
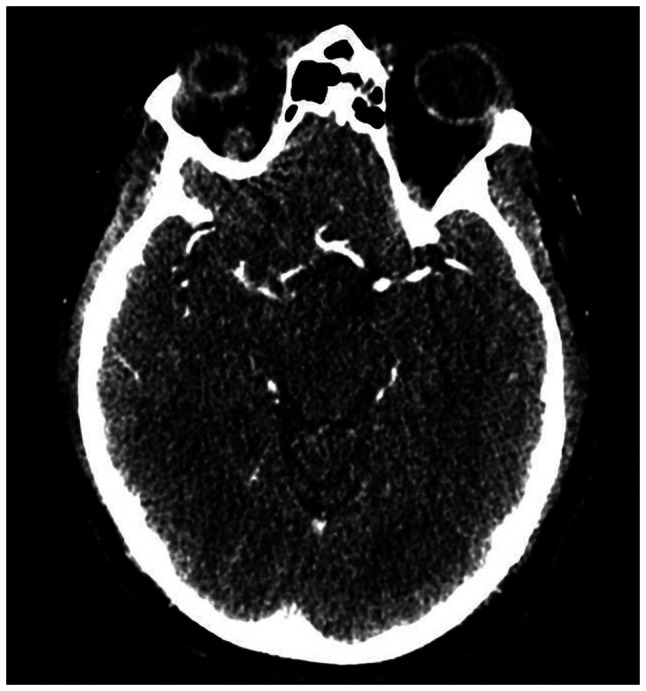
Computed tomography angiography scan.

**Figure 3:**
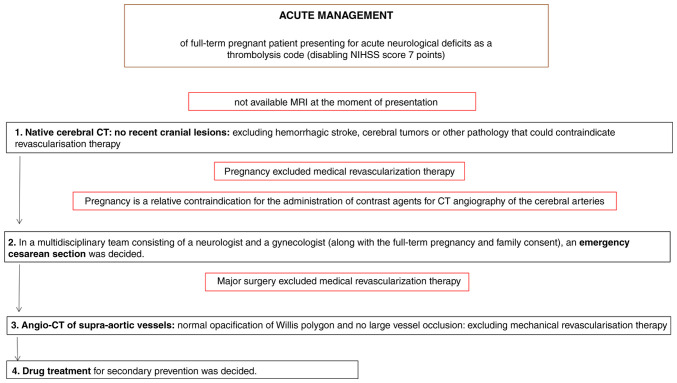
Diagram of the acute management of the pregnant patient with acute ischemic stroke in the revascularization window.

In terms of the diagnostic approach, in numerous developing countries, including Romania, a routine cerebral MRI is not commonly performed in emergency settings due to a combination of factors, such as limited healthcare resources and infrastructure challenges. Furthermore, Romania is still struggling to meet the European standards of quality in healthcare (11). The patient in the present case report was admitted during the night on a weekend; therefore, MRI was not available. As a result, the first radiological investigation was a native brain CT scan (intravascular contrast being avoided in pregnancy), which did not reveal any cranial lesions. The native cerebral CT scan in presented in Fig. 1. The clear native cerebral CT scan excluded other pathologies that would explain a sudden neurological deficit (such as a cerebral tumor) or that could contraindicate revascularization therapy (such as hemorrhagic stroke). Given the pregnancy, medical revascularization therapy with tissue plasminogen activator (tPA) was excluded. Moreover, as a CT scan with contrast or a CT angiography of the cerebral arteries were contraindicated in pregnancy, and as the patient was at 38 weeks of gestation with a viable fetus ready for delivery, a multidisciplinary team comprising a neurologist and an obstetrician recommended an emergency cesarean section. Although the procedure was planned for the following day, the urgency of the case and the consent of the family supported proceeding without delay. Following the delivery, the neurological deficits persisted, with a NIHSS score of 7 points. Furthermore, even though the patient was still in the thrombolysis window of 4.5 h following delivery, the major surgery contraindicated revascularization with tPA due to the massive hemorrhagic risk. However, the patient was still eligible for mechanical revascularization. Therefore, the patient underwent CT angiography of the cerebral arteries, which did not detect large vessel occlusion, and finally, mechanical thrombectomy was excluded and drug treatment for secondary prevention with aspirin was decided. The CT angiography of the cerebral arteries in presented in Fig. 2. The acute management of the patient is presented in Fig. 3.

The neonatal outcomes were favorable, with a birthweight of 2.840 g, an Apgar score of 9, with favorable neonatal adaptation and an increasing weight-growth curve. The mother demonstrated an excellent post-operative recovery: On post-operative day 1, the patient was mobilized, able to sit up and ambulate without assistance. The motor deficit was limited to the right upper limb (3/5 MRC). The patient reported no notable abdominal pain and vital signs remained stable throughout. Wound healing proceeded without complications, and the overall functional status of the patient improved, allowing the performance of routine maternal duties. By the end of the hospitalization period (from June 19 until June 30, 2023), the patient was able to feed the newborn with a bottle, even with a motor deficit in the right hand, demonstrating marked adaptability and resilience.

**Figure 4:**
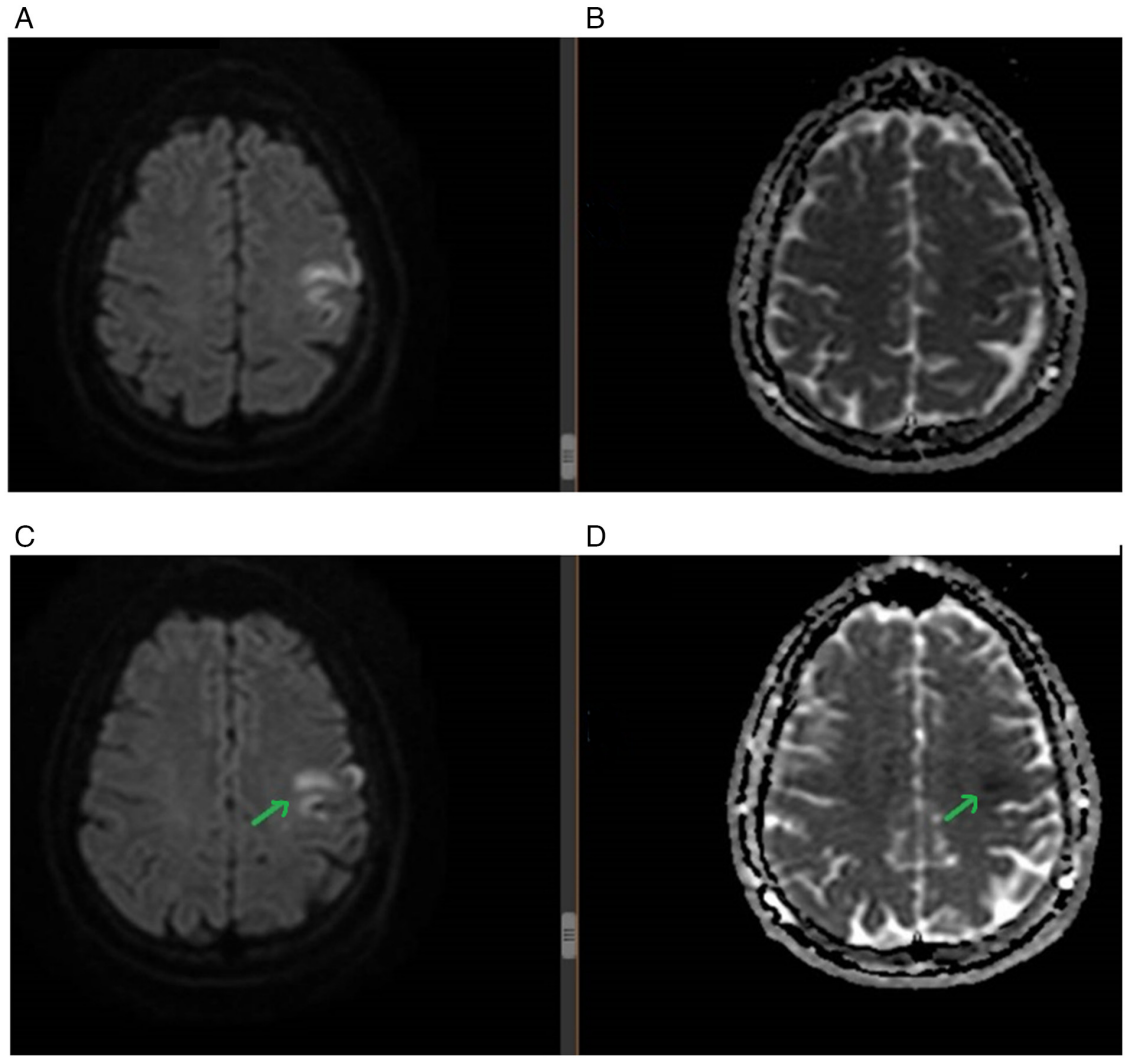
MRI findings. (A) Hyperintense lesion in diffusion sequence at the left frontal level. (B) Low apparent diffusion coefficient correspondence at the left frontal level. (C) Hyperintense lesion in diffusion sequence affecting the middle gyrus and precentral gyrus on the left side (green arrow). (D) Low apparent diffusion coefficient correspondence at the middle gyrus and the precentral gyrus on the left side (green arrow).

During admission, several paraclinical investigations were performed. A brain MRI revealed a hyperintense lesion in diffusion sequence with low apparent diffusion coefficient correspondence at the frontal level of the left side, affecting middle gyrus and precentral gyrus (Fig. 4). A Doppler ultrasound of cervico-cerebral vessels did not detect atheroma plaques. A 24-h electrocardiogram did not reveal atrial fibrillation episodes; however, an outpatient extended cardiac rhythm monitoring was recommended for 3-7 days. Microemboli detection revealed the passage of 8-10 microemboli, suggestive of an interatrial communication. A subsequent transesophageal ultrasound revealed a small patent foramen ovale (PFO), and an 8-point Risk of Paradoxical Embolism (RoPE) score of PFO was determined (no history of hypertension, no history of diabetes, stroke or cortical infarct, and an age of 29 years). This indicated an 84% chance that the cause of the stroke of the patient was due to PFO. However, the small anatomical proportions of the PFO did not indicate the need for interventional closure (12). Due to the PFO, a compression ultrasound of the lower limbs was performed, and no deep vein thrombosis was identified. Furthermore, vitamin B12 deficiency and mild anemia were detected.

However, laboratory tests for the usual hereditary thrombophilic risk factors for arterial embolism, such as factor V Leiden (G1691A), prothrombin G20210A, antithrombin deficiency, protein C and S deficiencies and antiphosolipid syndrome, were negative. The genotyping of the Factor V Leiden mutation was performed using a quantitative PCR assay (LightCycler^®^ Factor V Leiden Mutation Detection kit; cat. no. BM05, Roche Diagnostics) on the LightCycler^®^ 2.0 instrument, following the manufacturer s instructions. The prothrombin G20210A variant was analyzed by quantitative PCR using the LightCycler^®^ Prothrombin G20210A Mutation Detection kit (cat. no. BM06, Roche Diagnostics). Antithrombin functional activity was measured using a chromogenic assay (STA^®^ Antithrombin; cat. no. HE34, Diagnostica Stago, Inc.), performed on the STA-R Evolution analyzer. Protein C activity was assessed using a chromogenic method (Protein C Chromogenic assay; cat. no. HE35, Siemens Healthineers). Free Protein S antigen was quantified by immunoturbidimetry (Liatest^®^ Free Protein S; cat. no. HE36, Diagnostica Stago, Inc.). Testing for lupus anticoagulant followed the current International Society on Thrombosis and Haemostasis (ISTH) guidelines and included both screening and confirmatory assays: dRVVT screen and confirm reagents (LA1/LA2 dRVVT; cat. no. HE32, Werfen/Instrumentation Laboratory) and aPTT-based lupus-sensitive assay (SynthASil^®^; cat. no. HE32, Werfen). IgG and IgM anticardiolipin antibodies were measured using ELISA kits (QUANTA Lite^®^ ACA IgG/IgM; cat. nos. IM328, Inova Diagnostics, Inc.). IgG and IgM anti-β2GPI antibodies were determined using ELISA (QUANTA Lite^®^ β2-GPI IgG/IgM; cat. no. IM225, Inova Diagnostics, Inc.).

Building upon laboratory investigations suggesting a potential thrombophilic status, genetic testing revealed a homozygous mutation in the methylenetetrahydrofolate reductase (MTHFR) gene and heterozygous mutation in the plasminogen activator inhibitor-1 gene. The genotyping of MTHFR C677T and A1298C polymorphisms was performed using LightCycler^®^-compatible quantitative PCR assays with hybridization probes (LightMix^®^ kit MTHFR C677T/A1298C; cat. no. BM33, TIB MOLBIOL Syntheselabor GmbH). Reactions were run on the LightCycler^®^ 2.0 instrument following the manufacturer s protocol. The PAI-1 promoter 4G/5G polymorphism was detected using a LightCycler^®^-based quantitative PCR assay (LightMix^®^ kit PAI-1 4G/5G; cat. no. BM35, TIB MOLBIOL Syntheselabor GmbH), with allele discrimination performed by melting-curve analysis on the LightCycler^®^ 2.0 system. The results of thrombophilia screening and genetic thrombofilia testing are presented in Table I.

Due to the existing PFO with a high RoPE score and positive thrombophilic status, anticoagulation medication was decided for stroke secondary prevention, during hospitalization and in the first month after delivery with low molecular weight heparin, enoxaparin (80 mg, corresponding to 0.8 ml twice daily for a patient with a weight of 80-kg). Following this period, the patient received bridging therapy with both enoxaparin and acenocoumarol for 5 days. After these 5 days, oral anticoagulation with acenoumarol was dose-adjusted to maintain an international normalized ratio (INR) of 2.0-3.0. Finally, following adjustments, the patient remained on a dose of 2 mg of acenocoumarol daily.

## Discussion

The first particularity of the present case report is derived from the therapeutic management: The series of medical interventions when admitting the 38-week pregnant patient with acute ischemic stroke in the revascularization window. A native brain CT was initially performed to rule out another cause of acute neurological deficit, and after excluding cerebral tumors or hemorrhagic stroke, an emergency cesarean section was performed under general anesthesia. Subsequently, a CT angiography of the cerebral arteries was performed. Finally, a mechanical revascularization procedure would have followed if a large vessel occlusion was identified.

The first factor that should highlight potential thrombolysis in pregnant patients is stroke severity: NIHSS score, stroke position, consciousness, motor and sensitive deficits, language disorder and speech disorder (1). The second factor that should be taken into account is the risk of bleeding, considering previous pathologies of the patient, such as the bleeding history, hypertension and other known coagulopathies (1). Personal factors should also be taken into account, such as age, medical history and body weight (1). Lastly, from an obstetrical point of view, the decision to thrombolyze a pregnant patient should consider gestational age and obstetrical conditions (1). Other previous personal pathologies may increase the risk of stroke in pregnancy, such as: Hematologic diseases (sickle cell anemia, thrombocytopenia and thrombophilia), cardiac disease (hypertension and heart disease), diabetes and migraine-type headaches (6). Other risk factors include smoking and alcohol consumption (5,13).

The second particularity of the present case originates from the diagnosis of acute ischemic stroke in a full-term pregnant patient, and the evaluation the possible etiologies of cryptogenic stroke. Therefore, the present case report emphasizes the importance of evaluating thrombophilic status and PFO in patients with ischemic stroke at a young age. The most probable etiology of the stroke was the procoagulant status of the patient based on the pregnancy itself, the peripartum period of the patient and the positive genetic thrombophilic status. Notably, the patient had no personal history pointing towards this diagnosis, such as history of spontaneous abortions, superficial or deep venous thrombosis, or pulmonary thromboembolism.

It has been suggested that PFO is an independent risk factor for ischemic stroke, particularly in young patients with cryptogenic stroke; however, the causality between PFO and ischemic stroke is not yet confirmed (14,15). Moreover, when patient suffering a stroke is diagnosed with PFO, this is not an indication that PFO was the cause of the stroke; therefore, secondary prevention for these patients is still debatable (14). However, in the event that a patient suffering a stroke is discovered with both PFO and prothrombotic coagulopathies, oral anticoagulants should be considered for secondary prevention, rather than platelet inhibitors (14). Other studies have suggested that warfarin is superior to platelet inhibitors as a secondary prevention for PFO-related stroke cases (16,17).

Moreover, the placement of infarct localization of a PFO-related stroke is due to the physiopathological mechanism of a PFO: As they are an interatrial shunt, they will allow smaller emboli to pass through the PFO (left-right) and then reach multiple scattered places within the brain on both hemispheres (18). Thus, in terms of imaging characteristics, PFO-related stroke is usually revealed as a single cortical ischemic lesion or multiple small scattered ischemic lesions (18).

For PFO-related strokes, thrombogenic conditions are usually present, as they lead to a predisposition of venous thrombus formation and further lead to a paradoxical embolism (18). Pezzini *et al* (14) reported that the G20210A variant of the prothrombin gene and the G1691A mutation of factor V gene had a higher rate of prevalence in patients with stroke and PFO, suggesting a possible pathophysiological role of these minor thrombophilias. By contrast, their study also reported that the homozygous deficient TT MTHFR genotype was not a genetic minor thrombophilia found in patients with PFO-related stroke (14). Furthermore, a recent study reported that usual risk factors for stroke (such as smoking, dyslipidemia or diabetes) are not associated with risk for PFO-related stroke (19). Moreover, that study highlighted that thrombophilias or intracardiac shunt size are not associated with stroke occurrence in patients with PFO (19).

Lastly, the third particularity of the present case comes from the medication treatment. From a neurological perspective, secondary prevention following cryptogenic stroke is generally achieved with aspirin or a direct oral anticoagulant. A total of two large randomized trials, NAVIGATE ESUS and RE-SPECT ESUS, evaluated the efficiency of rivaroxaban and dabigatran, respectively, compared with aspirin; however, the results demonstrated no definitive superiority of direct oral anticoagulants over aspirin, and the evidence remains under evaluation (19-21). However, for the patient in the present case report, with both PFO and positive thrombophilic status, anticoagulation was decided as secondary prevention: Considering the postpartum status of the patient, low molecular weight heparin was administered for the first month following delivery, and acenocoumarole was then administered for the ensuing 3 months.

Furthermore, from a psychological perspective, following ischemic stroke, young adults are more predisposed to fatigue, depression and anxiety (23,24). Other feelings may include anger, denial, frustration, negative body image and impaired self-esteem (24). Additionally, certain common practical problems that young stroke survivors experience are family conflicts, loss of home, loss of employment and loss of spouse (25), with psychological adjustments including reduced quality of life (associated with dependence, being single and unemployment), financial stress, conflicts with spouses, children, childcare difficulties, sexual problems, separation, reduced social and leisure activities, disruption of self and identity and reduced life satisfaction (24). It has been suggested that depot injectable medication may increase quality of life in patients with psychotic symptoms (26). Furthermore, pregnancy and the peripartum period is considered a critical period for marriage, and pregnant women are at risk for developing marital burnout (27). Burnout syndrome is characterized by psychiatric, psychosomatic, somatic and social symptoms; whilst chronic fatigue, continuous exhaustion and mental stressors may arise during pregnancy and peripartum; therefore, this is a period where women are predisposed to burnout syndrome (28). Medical treatment for psychiatric symptoms in pregnant patients or patients during the peripartum period should be carefully considered and given only if the benefits exceed the risks for the infant.

In conclusion, the particularity of the case described herein originates from the series of medical interventions in a full-term pregnant patient who presented with acute ischemic stroke in the revascularization window: Clinical examination, native head CT scan, emergency cesarean section, CT angiography of the cerebral arteries, and, eventually, mechanical thrombectomy, were performed. The present case report also emphasizes the importance of evaluating inherited genetic thrombophilia and PFO in young patients suffering a stroke, and highlights the need for psychological and psychiatric evaluation for possible reactive depression, anxiety and burnout in young patients suffering a stroke, particularly in the peripartum period.

## Figures and Tables

**Table I:** Thrombophilia status results.

Laboratory test	Result	Reference interval
Thrombophilia screening		
Factor V Leiden with activated protein C	91.1	38-144/sec
Factor V Leiden without activated protein C	32.2	24-184/sec
Activated protein C ratio	2.83	≥2.3
Antithrombin deficiency	142	83-128%
Protein C deficiency	135	70-140%
Protein S deficiency	60.4	54.7-123.7%
Lupus anticoagulant screen	38.5	29-39.76
Lupus anticoagulant confirm	28.9	27.6-36
Homocisteinemia	7.65	4.3-11.1/µmol/l
Genetic thrombophilia testing		
MTHFR gene: A1298C mutation	Homozygous genotype	Negative
MTHFR gene: C677T mutation	Negative	Negative
PAI-1 gene (polimorfism 675 4G/5G)	Heterozygote genotype	Negative
Factor V Leiden mutation	Negative	Negative
Factor II (prothrombin) mutation	Negative	Negative

## Data Availability

The data generated in the present study may be requested from the corresponding author.
